# In-Hospital Patient Safety Events, Healthcare Costs and Utilization: An Analysis from the Incident Reporting System in an Academic Medical Center

**DOI:** 10.3390/healthcare8040388

**Published:** 2020-10-07

**Authors:** Yao-Wen Kuo, Jih-Shuin Jerng, Chia-Kuei Lin, Hsiao-Fang Huang, Li-Chin Chen, Yu-Tzu Li, Szu-Fen Huang, Kuan-Yu Hung

**Affiliations:** 1 Department of Integrated Diagnostics & Therapeutics, National Taiwan University Hospital, Taipei 10002, Taiwan; kyw@ntu.edu.tw; 2 Department of Internal Medicine, National Taiwan University Hospital, Taipei 10002, Taiwan; kyhung@ntu.edu.tw; 3 Center for Quality Management, National Taiwan University Hospital, Taipei 10002, Taiwan; linck@ntuh.gov.tw (C.-K.L.); 105307@ntuh.gov.tw (H.-F.H.); lichin@ntuh.gov.tw (L.-C.C.); 102112@ntuh.gov.tw (Y.-T.L.); sfhuang0406@ntuh.gov.tw (S.-F.H.)

**Keywords:** patient safety events, incident reporting, healthcare cost, utilization

## Abstract

The possible association of patient safety events (PSEs) with the costs and utilization remains a concern. In this retrospective analysis, we investigated adult hospitalizations at a medical center between 2010 and 2015 with or without reported PSEs. Administrative and claims data were analyzed to compare the costs and length of stay (LOS) between cases with and without PSEs of the three most common categories during the first 14 days of hospitalization. Two models, including linear regression and propensity score-matched comparison, were performed for each reference day group of hospitalizations. Of 14,181 PSEs from 424,635 hospitalizations, 69.8% were near miss or no-harm events. Costs and LOS were similar between fall cases and controls in all of the 14 reference days. In contrast, for cases of tube and line events and controls, there were consistent differences in costs and LOS in the majority of the reference days (86% and 57%, respectively). Consistent differences were less frequently seen for medication events and control events (36% and 43%, respectively). Our study approach of comparing cases with PSEs and those without any PSE showed significant differences in costs and LOS for tube and line events, and medication events. No difference in cost or LOS was found regarding fall events. Further studies exploring adjustments for event risks and harm-oriented analysis are warranted.

## 1. Introduction

The context and practice of clinical care have improved in recent years in terms of patient safety through advances in medicine and technology. However, the occurrence of adverse events (AEs), defined as an injury caused during the health care process, rather than by the underlying disease or condition of the patient, remains a significant issue [1,2,3,4]. The Institute of Medicine reported that medical errors cause more than a million injuries annually in the USA [5], and that patient harm occurs in 25.1% admissions. Moreover, about half of these cases required intervention or caused permanent harm, and 63.1% were regarded as being preventable [6]. 

Promoting patient safety usually involves a common strategy to detect patient safety events (PSEs), defined as an unexpected or unintended event that could have led or did lead to harm to the involved patient during the care process. Typical events might include patient falls; medication events such as prescription errors, dispensing errors, and drug administration errors; tube and line events such as inadvertent removal of an indwelling tube or line, etc. With this approach, studies have estimated an AE rate of 2.9–16.6% [6], prompting the establishment of clear processes to detect, aggregate, and analyze PSEs to generate strategies to prevent the occurrence of PSEs, improve care quality, and promote a better patient safety climate [7]. Typical endeavors by the healthcare institutions emphasize the establishment of an incident reporting system (IRS) and encourage reporting [8,9], while others have also encouraged analyzing PSEs to gain insights into systemic problems and preventive measures [10,11,12].

Besides, the association between PSEs and healthcare utilization and costs has also been increasingly studied. A study in the period 2003–2004 reported the AE-related costs of over two billion Australian dollars annually [13]. As there has been a trend of non-reimbursement for AE-derived medical expenses [14,15,16], cost estimation and analysis of its structure has become increasingly important. Reports regarding AE-related costs commonly analyze events identified from computerized medical records, payment claims with diagnosis coding, or global trigger tools, while analyses have usually focused on one or a few event categories, such as falls.

Among the methods used to analyze PSE-related costs [17], linear regression is the conventional method [18]. However, this method might ignore the interactions between hospital length of stay (LOS) and the occurrence of events that may have a reciprocal causal relationship. Propensity score-matching (PSM) with comparisons has also been advocated [19], which provides insight into cohort observations under randomization and includes time as an essential factor. Nevertheless, reports applying and comparing PSM with other methods in analyzing PSE-related costs are limited. The purpose of this study was to apply these two methods to analyze the healthcare costs and utilization associated with the occurrence of inpatient PSEs.

The remainder of the paper is organized as follows. The next section outlines the methodology of this study. We subsequently present and discuss our findings and provide concluding remarks.

## 2. Materials and Methods 

### 2.1. Study Design and Setting

This retrospective study was conducted at National Taiwan University Hospital to analyze the cost and healthcare utilization associated with PSEs between 1 January 2010, and 31 December 2015. The Institutional Research Ethics Committee of the National Taiwan University Hospital approved this study (#201709046RINA) and waived the need for informed consent from the patients. 

The hospital is a medical center, with about 2300 beds and approximately 6800 workers, including 460 attending physicians, 650 residents, 2800 nurses, and other workers. The hospital’s Incident Reporting System (IRS) was established in 2002; all of the PSE reports in this study were provided electronically. Staff from the Center for Quality Management of the hospital validated the content, patient harm, and severity of each PSE after receiving the report.

### 2.2. Participants

Patients admitted to the hospital during the study period were included. Patients with at least one reported PSE were labeled as PSE cases; the remaining hospitalizations without any reported PSE were classified as controls. This labeling was used to retrieve relevant clinical, administrative and claims data upon request from the Integrated Medical Database (NTUH-iMD) of the Department of Research of the National Taiwan University Hospital, who provided de-identified data for research purposes. Patients were excluded if they had at least one of the following conditions: (1) admitted for end-of-life management; (2) admission not paid by the National Health Insurance program.

A *patient safety event* (PSE) was defined as an unexpected or unintended event, which could have led to or did lead to harm to the involved patient. These events consisted of the following types: an *adverse event* (AE) was defined as an injury caused during the health care process rather than by the underlying disease or condition of the patient; a *no-harm event* was defined as an event which resulted in no harm to the patient, or the effect was so minor that the patient could not even feel it; a *near miss event* was defined as an event that may have caused an accident, injury or illness, but did not due to an unintentional or timely intervention [20].

### 2.3. Variables

To analyze the association of PSEs with costs and healthcare utilization, the investigators acquired the following variable: demographic data, admitting department, category of event, the severity of harm to the patient, claimed medical expenses of the hospitalization, and length of stay.

### 2.4. Data Sources, Collection, and Measurements

The following data were collected for each PSE from the IRS database: age of the patient, department, and ward for admission, category of events resulting in the PSE, severity of harm to the patient. Furthermore, after the approval by the Research Ethics Committee, the study investigators requested the Integrated Medical Database (NTUH-iMD) of the Department of Research of this hospital to providing de-identified clinical and administrative data. The database provided cases with de-identification data for research purposes. Patient-level costs were obtained from the claims for each admission for the reimbursement by the National Health Insurance of the Taiwan, which provided universal healthcare coverage of the patients and set the prices for hospital care. Costs were converted into US dollars based on the exchange rate toward the end of 2015. The inflation rate in Taiwan has been maintained at a steady low level, about 1.09% annually; therefore, the authors decided that the inflation rate would not be included in the adjustment. The primary outcome measures were total financial costs based on the claims and length of stay.

### 2.5. Bias and Study Size

Since the under-reporting of PSEs remains a commonly understood issue across the institution, the study might encounter *selection bias* to assigning patients with PSE to the control group because of the non-reporting of events. This situation could also result in *information bias*, in that the control group could also suffer from unreported PSEs. These types of bias could result in underestimation of the impact of costs and LOS caused by the PSEs.

As the investigators were not able to have a reference cost estimate from a pilot analysis in the same hospital or a similar study from another institution, the study size was not estimated, and the investigators decided that all of the reported PSE in the IRS would be analyzed after proper inclusion and exclusion processes were applied.

### 2.6. Statistical Analysis

We first performed a descriptive analysis of the included reported PSEs regarding their category, severity, and time of occurrence concerning the admission date to show the general background scenario. Categorical data are expressed as number (%). Continuous variables were expressed as mean ± SD and 95% confidence interval (95% CI). We then performed inference analysis to compare the direct costs and LOS between the cases and controls according to the model used. We then selected the most common categories of PSE, together contributing to at least 50% of the PSEs. In this study, we used two models to estimate differences in the direct costs and LOS between the cases and controls. In both models, we only selected the cases with a reported PSE within the first 14 days of hospitalization as the cases and then stratified these cases into 14 groups based on the day when the PSE was reported. The control cases were those who did not have any reported PSE and were not discharged until at least the stratified day. For example, for the reported PSEs that occurred on the third day after admission, we only included controls with an LOS of at least three days. 

In the first model, we performed linear regression analysis to estimate direct costs and LOS, including age, gender, diagnosis, Charlson comorbidity index [21,22], and the main department where the care was provided. In the second model, the propensity score was the conditional probability for having a PSE as a binary dependent variable. Age, gender, main diagnosis, Charlson comorbidity index, admission department and calendar year of admission were added into a non-parsimonious multivariable logistic regression model to predict the effect of PSEs. The propensity score for each individual was the predicted probability derived from the logistic equation. We used PSM to compare the costs and LOS between the groups with and without PSEs. The estimated propensity scores from the datasets were then combined using the mean of the individual estimates in each dataset, according to Rubin’s rule [23]. One-to-one matching by propensity score, without replacement, was performed using the nearest neighbor method within a caliber of 0.1. We performed matching separately within each event category. PSM was performed using SAS software (version 9.4, SAS Institute Inc., SAS Campus Drive, Cary, North Carolina, USA). The matched cases were then identified, and comparisons were made between cases and controls. 

Continuous variables were expressed as mean ± SD. Statistical analyses were performed using SAS software (SAS Institute Inc., USA). All statistical comparisons were two-tailed. A *p*-value < 0.05 was considered to indicate statistical significance.

## 3. Results

### 3.1. Demographic Data

During the 6-year study period, there were 424,635 admissions to the hospital. Table 1 summarizes the demographic features of the admitted patients. Neoplasms accounted for the most common diagnosis, but only accounted for 23.4% of the patients. Patients admitted to the Departments of Surgery and Internal Medicine accounted for nearly half of the cases.

### 3.2. Patient Safety Events

A total of 14,181 PSEs related to hospitalized patients during the study period were reported to the IRS, with an incident rate of 3.3 per 100 admissions. Table 2 summarizes their features. Of these events, 1598 (11.3%) were near misses, 8301 (58.5%) were no-harm events, 3578 (25.2%) had mild harm to the patients, 424 (3.0%) had moderate harm, 34 (0.2%) had severe harm and 66 (0.5%) had very severe harm or death. We chose the three most common categories related to patient care processes for further analysis, including falls (26.4%), tube and line events (18.8%), and medication events (15.9%). Fall events included all hospitalized patients with a fall accident. Tube and line events included inadvertent removal of tubes or lines, loss of function, malpositioning, and complications related to the placement of tubes and lines other than for infection, such as bleeding and dislodgement. Medication events included those related to the prescription, dispensing, and administration of medications. Administrative events included registration and appointment errors that would lead to delayed or cancelled care processes. There appeared to be a trend toward fewer PSEs through the study period, with the numbers of PSEs of being 2306, 2434, 2855, 2336, 2337, and 1913 in each of the six years from 2010 to 2015 (Table 2). PSEs occurring during the first 14 days of hospitalization accounted for a substantial proportion of all PSEs in the three most commonly reported categories, including falls (45.3%; *n* = 1695), tube and line events (38.4%; *n* = 1024), and medication events (42.5%; *n* = 958). Therefore, we decided to use the first 14 days of hospitalization in these three categories as the analysis cohort.

### 3.3. Inclusion and Selection for Difference Analysis

We further analyzed differences in the costs and LOS for the three most common PSEs, including falls, tubes and lines, and medication events. We then selected the three most common categories (falls, line and tube events, and medication events) of PSE that together contributing to at least 50% of the PSEs. As a result, we chose three of the most commonly reported categories of PSEs for inference analysis based on the rationale that the occurrence of surgical and transfusion events should only develop in those patients who had received or at least been arranged for these two categories of treatment. As our study data lacked a detailed daily list of surgical procedures and the amount of transfusion, adjustment on these factors before comparison by each reference day was not possible. To estimate the differences in direct hospital costs and LOS, we selected PSEs within 14 days of hospitalization and control hospitalizations without PSEs. Table 3 shows the number of selected PSEs according to the day they occurred after hospitalization and the number of selected controls with an LOS of at least from admission to the index day. In the multivariable linear regression model (model 1), all of the PSEs in all three categories were included and analyzed by the reference day. In PSM (model 2), the number of PSE cases was slightly reduced for some reference days after matching, whereas the number of non-PSE controls corresponded with the PSE cases. PSEs most commonly occurred on day 2 of hospitalization (Table 3).

### 3.4. Differences in Cost and Length of Stay for Different Types of Events

#### 3.4.1. Differences in Cost and LOS for Fall Events

Table 4 shows the results of logistic regression and PSM to estimate differences in costs and LOS between cases with a fall event and non-PSE controls. For most of the groups divided by reference day, there were no significant differences in cost and LOS between the cases and controls. Only one reference day (day 12) in the PSM model showed a difference in LOS (Table 4).

#### 3.4.2. Differences in Cost and LOS for Tube and Line Events

Table 5 shows the results of logistic regression and PSM to estimate differences in costs and LOS between the cases with a tube or line event and non-PSE controls. For most of the groups divided by reference day, more than half of the reference days had consistent significant differences in costs (86% of the reference days) and LOS (57% of the reference days) between the cases and controls (Table 5). PSM model found fever reference day groups with a more significant difference in costs and LOS than the linear regression model.

#### 3.4.3. Differences in Cost and LOS for Medication Events

Table 6 shows the results of logistic regression and PSM to estimate differences in costs and LOS between cases with a medication event and non-PSE controls. For most of the groups divided by reference day, the logistic regression model showed consistent significant differences in the cost and LOS between the cases and controls. However, PSM showed fewer groups by reference day with significant differences in costs and even fewer day groups with significant differences in LOS. Therefore, the consistent difference was found in only 36% (5 of 14) of the reference days for costs and 43% (6 of 14) of the reference days for LOS between the two groups (Table 6). 

As can be seen in Table 4, Table 5 and Table 6, while the cost differences were not necessarily estimated to be higher by either LOS model or PSM model, the LOS differences appeared to be estimated higher by the PSM model, counting those reference days with statistical significance in the differences.

## 4. Discussion

Our results showed that of the hospitalizations with and without PSEs during the first 14 days of admission, the extents of differences in costs and LOS varied across the three PSE categories. Differences in costs and LOS for fall events were not significant by either of the two comparison models; however, the differences were more consistently significant for tube and line events and were less consistently significant for medication events by the two estimation models. A feature of this paper is the novel approach with the reference day-stratified grouping of cases against controls based on the occurrence or absence of patient safety events. We believe that this methodology has not been reported in the literature. We also employed two analysis methods, including linear regression and propensity score-matched comparison, and the results were similar between the two methods. The comparison between these two methods based on reference day stratification approach has not been reported before, either.

The comparisons of costs and LOS in this study were based on the presence or absence of reported PSEs through the institutional IRS; thus, in addition to AEs, the analyses also included events without associated patient harm and those reported for identified undesired processes, materials, equipment and facilities related to patient care. Therefore, this study provides a broader approach to the understanding of undesired contexts of care concerning the burden of healthcare systems. A study showed that AE-related additional financial costs were highest for surgery-related events, with an additional 10.9 hospital days and additional costs of USD 57,727, while an event occurring at medical wards led to an additional 9.8 hospital days and an additional cost of USD 38,656 [24]. Another study reported that AEs resulted in an average increase of 2.2 hospital days and an increased cost of USD 3244, with 31.6% of the events being preventable and contributing to 4.6 of hospital days and USD 5857 of related costs [25]. Our study, despite a broader approach, including PSEs without patient harm, still showed significant financial and utilization impacts, except for the category of fall events. This finding suggests that PSEs are at least a marker of increased cost and utilization of healthcare for hospitalizations.

Our claims-data-based approach to estimate costs might have ignored other hidden costs such as administration costs in terms of reporting, data collection, meeting and discussion, improvement activities, and maintenance of the facilities and materials related to health care. Although the cost difference was not significant for fall events, the aggregated cost may still have had a large impact on healthcare. However, standardized methods to assess the burden related to PSEs are currently lacking [17]. Although the data sources studies accessed might include claim database [26], full cost-benefit/utility evaluations are rarely completed as they are resource-intensive and often require unavailable data [27]. We used two models to compare differences in costs and LOS, including multivariable linear regression and PSM. This study employed the reference days as the basis of grouping for comparison, which might exert control of these two variables (features) by assuming the PSE cases and control cases had similar costs until the reference day because of the same in-hospital stay. As mentioned in the Introduction, we were worried about the soundness of linear regression that this method might ignore the interactions between hospital length of stay (LOS) and the occurrence of events that may have a reciprocal causal relationship. Therefore, we proposed a reference-day-based PSM comparison to understand the robustness of the model. The variables included in these models were limited; therefore, we were not able to exclude interference by other factors such as the severity of illness and interventions, which may vary widely, despite a large number of control cases in this study. As can be seen in Table 4, Table 5 and Table 6, while the cost differences were not necessarily estimated higher by either LOS model or PSM model, the LOS differences appeared to be estimated to be higher by the PSM model, counting those reference days with statistical significance in the differences. The literature lacked a previous report of the comparison between these two models in similar studies regarding the impact of PSE or AE on costs and LOS; therefore, further studies are required to elucidate whether these two models do show discrepancy in results. Additional experiments have to be carried out to validate the feasibility and robustness of the proposed models.

Given the higher financial burden and longer LOS, our study may not imply causality between the occurrence of PSEs and higher costs. Specific categories of PSE, such as tube and line and medication, would not occur if the patients do not receive the specific materials for the intervention. The complexity of a clinical condition might also be associated with the materials used, and intervention received, contributing to a higher cost and carrying a higher risk of PSE. Therefore, the cost and LOS may have the same risk factors as the cases with PSEs. Moreover, as most of the patients with PSEs did not suffer from any harm, a longer LOS is less likely to be caused by the PSE, but may be related to the disease and the care provided. Another possible explanation is that the occurrence of a PSE, even if it did not result in patient harm, may be linked to an undesired quality relating to the care process and undesired context. This situation may also have affected the effectiveness and efficiency of care, as reflected by increased costs and prolonged LOS. Further studies to comprehensively investigate the financial impact associated with these events are needed. 

### Limitations

There are several limitations to this study. First, reporting PSE was voluntary; therefore, underreporting of PSEs is highly probable, as has been reported previously [28]. However, we do not know whether this led to an over- or underestimation of the associated costs and LOS. Supplementary methods, such as trigger tools and audits, may be useful [29]. Second, not all of the PSEs may have been related to patient harm, such as equipment defects and undesired working conditions, and the actual process of care may not have been affected. Third, estimations were not adjusted for disease severity; therefore, we did not know whether the patients who experienced PSEs were more ill than the control group patients and whether that also contributed to higher medical expenses related to the disease and routine care rather than to the events themselves. Fourth, we did not use purchasing power parity for converting the costs to US dollars, which might be more appropriate than exchange rates. Fifth, we chose not to compare costs and LOS between cases with patient harm and those without PSEs, mainly because the number of cases with patient harm was too small, with less than one-third of cases involving harm and less than 5% moderate or more severe harm. The number of cases would have been even smaller if they were separated by the day of occurrence, and this may have reduced the statistical power of the analyses. Larger scale analysis is warranted. Based on these limitations, we hypothesize that the negative impact of financial costs on healthcare systems may be much higher than estimated in this study. We believe that the occurrence of PSEs during admission is a marker of high cost for care services. Last, our study chose the reference-day-stratified comparison of groups of patients without any PSE before the reference day. Therefore, competing risk analyses were not performed in this study, and we do not know if patients with repeated PSEs of the same or different event category had a similar pattern of cost and LOS differences. Our findings, therefore, require further validation.

## 5. Conclusions

In conclusion, we aimed to analyze the difference in cost and length of stay between admission with PSE and without one. Our findings showed mixed results for the differences in costs and length of stay between admission with PSE and without. The differences in costs and LOS were not significant in cases with fall event and those without PSE, while the costs and LOS differences were more significantly different in terms of tube and line events and medication events. We employed reference day-based analysis with propensity score-matched comparison to provide a possible method for the estimation of PSE-associated cost and LOS. This method might provide a deeper understanding of the impact of PSEs on the healthcare burden. Further studies exploring adjustments for event risks and harm-oriented analysis are warranted.

## Figures and Tables

**Table 1:** Demographics of the study population (*n* = 424,635).

Variable	*n* (%)
Age, mean ± SD	49.42 ± 24.52
Age, median (IQR)	54 (35–67)
Charlson Comorbidity Index, mean ± SD	1.72 ± 2.60
Charlson Comorbidity Index, median (IQR)	0 (0–2)
Male	211,551 (49.8)
Main diagnosis	
Neoplasms	98,104 (23.4)
Diseases of the circulatory system	37,155 (8.9)
Diseases of the digestive system	32,656 (7.8)
Diseases of the respiratory system	27,931 (6.7)
Injury and poisoning	25,991 (6.2)
Diseases of the genitourinary system	24,187 (5.8)
Diseases of the musculoskeletal system and connective tissue	19,638 (4.7)
Diseases of the nervous system and sense organs	18,261 (4.4)
Complications of pregnancy, childbirth, and the puerperium	17,288 (4.1)
Endocrine, nutritional and metabolic diseases, and immunity disorders	8929 (2.1)
Symptoms, signs, and ill-defined conditions	8640 (2.1)
Congenital anomalies	7979 (1.9)
Diseases of the skin and subcutaneous tissue	7962 (1.9)
Mental disorders	4780 (1.1)
Diseases of the blood and blood-forming organs	2727 (0.7)
Certain conditions originating in the perinatal period	1786 (0.4)
Infectious and parasitic diseases	1622 (0.4)
*Supplementary Materials classification of factors influencing health status and contact with health services*	72,951 (17.4)
Department	
Surgery	96,388 (22.7)
Internal Medicine	93,120 (21.9)
Pediatrics	47,218 (11.1)
Obstetrics & Gynecology	38,683 (9.1)
Orthopedics	28,217 (6.7)
Oncology	26,975 (6.4)
Urology	27,555 (6.5)
Otolaryngology	17,603 (4.2)
Ophthalmology	12,631 (3.0)
Traumatology	8490 (2.0)
Family Medicine	5826 (1.4)
Dentistry	5188 (1.2)
Psychiatry	4688 (1.1)
Neurology	4582 (1.1)
Physical Medicine & Rehabilitation	3680 (0.9)
Dermatology	2741 (0.7)
Gerontology	344 (0.1)
Others	578 (0.1)
Cost *, mean ± SD	3210 ± 7120
Cost *, median (IQR)	1479 (765–3122)
Length of stay, day, mean ± SD	10.4 ± 22.8
Length of stay, day, median (IQR)	5 (3–10)

* Converted to US dollars based on the exchange rate toward the end of 2015.

**Table 2:** Summary of category and severity of patient safety events (*n* = 14,181).

Variable	*n* (%)
Event category	
Fall	3741 (26.4%)
Tube and line *	2664 (18.8%)
Medication	2256 (15.9%)
Surgery	1360 (9.6%)
Transfusion	1139 (8.0%)
Examination	535 (3.8%)
Security	337 (2.4%)
Medical care	290 (2.0%)
Violence	261 (1.8%)
Cardiopulmonary arrest	250 (1.8%)
Administrative **	193 (1.4%)
Public space event	141 (1.0%)
Chemotherapy	137 (1.0%)
Facility and devices	97 (0.7%)
Anesthesia	13 (0.1%)
Other	767 (5.4%)
Severity	
Near miss	1598 (11.3%)
No harm event	8301 (58.5%)
Mild harm	3578 (25.2%)
Moderate harm	424 (3.0%)
Severe harm	34 (0.2%)
Very severe harm	2 (0.01%)
Death	64 (0.5%)
Year	
2010	2306 (16.3%)
2011	2434 (17.2%)
2012	2855 (20.1%)
2013	2336 (16.5%)
2014	2337 (16.5%)
2015	1913 (13.5%)

* Including inadvertent removal of tubes or lines, loss of function, malpositioning, and complications related to the placement of tubes and lines other than for infection, such as bleeding and dislodgement. ** Including registration and appointment errors that would lead to delayed or cancelled care processes.

**Table 3:** Number of cases and controls (without events) of the analyses.

Model 1: Multivariable linear regression				
**Reference Day**	**Without Events**	**Fall**	**Tube and Line**	**Medication**
1	413,252	167	69	174
2	374,101	285	132	161
3	292,667	202	143	104
4	234,403	179	118	99
5	196,314	142	110	65
6	165,600	156	97	80
7	142,239	129	76	59
8	124,142	110	55	59
9	108,335	85	74	37
10	97,049	93	59	46
11	87,628	85	44	46
12	79,463	62	47	28
13	72,227	75	49	29
14	66,045	63	45	34
Model 2: Propensity score matching				
**Reference Day**	Without Events *	**Fall**	**Tube and Line**	**Medication**
1	Same as event	166	68	174
2	Same as event	285	132	162
3	Same as event	203	144	103
4	Same as event	176	118	97
5	Same as event	139	110	62
6	Same as event	155	96	80
7	Same as event	126	76	58
8	Same as event	109	55	56
9	Same as event	86	70	36
10	Same as event	93	59	45
11	Same as event	87	44	45
12	Same as event	61	46	28
13	Same as event	75	49	29
14	Same as event	63	45	34

* The numbers of matched cases without an event were the same as the events matched for each type of patient safety events.

**Table 4:** Differences in cost and length of stay for fall events (reference: without event).

Difference in cost						
	Multivariable Linear Regression			Propensity Score Matching		
**Day**	**Estimate**	**SE**	***p*-value**	**Estimate**	**SE**	***p*-value**
1	−527.4	447.0	0.238	109.7	490.1	0.823
2	−445.1	354.9	0.210	−210.8	605.5	0.728
3	−324.6	467.4	0.487	−3.9	468.7	0.993
4	−67.0	545.0	0.902	865.7	535.4	0.107
5	−916.7	654.2	0.161	−180.6	529.9	0.734
6	−1183.1	672.6	0.079	−39.9	559.9	0.943
7	−1111.1	790.2	0.160	−1273.9	1035.1	0.220
8	−77.7	900.3	0.931	184.5	1096.2	0.867
9	−1383.8	1088.7	0.204	−1475.0	1105.8	0.184
10	432.3	1064.9	0.685	1451.5	1104.2	0.190
11	−1005.0	1157.5	0.385	653.8	1250.7	0.602
12	1291.5	1415.9	0.362	2250.7	1888.1	0.236
13	−776.2	1320.9	0.557	−868.3	1331.8	0.515
14	−893.9	1493.1	0.549	1209.6	1367.3	0.378
Difference in length of stay						
	Multivariable Linear Regression			Propensity Score Matching		
**Day**	**Estimate**	**SE**	***p*-value**	**Estimate**	**SE**	***p*-value**
1	0.5	1.1	0.656	1.9	1.7	0.273
2	−0.3	0.8	0.688	0.9	1.3	0.493
3	−0.4	1.1	0.704	0.9	1.3	0.488
4	0.9	1.3	0.459	2.0	1.8	0.276
5	−0.5	1.5	0.755	−0.3	2.7	0.905
6	−1.9	1.5	0.220	2.3	1.9	0.222
7	−0.8	1.8	0.648	−1.8	2.8	0.522
8	1.5	2.0	0.468	2.6	2.9	0.375
9	−1.3	2.4	0.585	−2.1	3.6	0.569
10	2.3	2.4	0.338	4.4	2.9	0.138
11	−0.8	2.6	0.749	3.2	2.8	0.262
12	0.9	3.2	0.776	5.6	2.5	0.025 *
13	−1.7	3.0	0.569	1.2	2.4	0.621
14	−1.3	3.3	0.703	2.0	3.0	0.508

* *p* < 0.05.

**Table 5:** Differences in cost and length of stay for tube and line events (reference: without event).

Difference in cost						
	Multivariable Linear Regression			Propensity Score Matching		
**Day**	**Estimate**	**SE**	***p*-value**	**Estimate**	**SE**	***p*-value**
1	4542.6	696.2	<0.001 ***	3635.6	1724.2	0.037 *
2	2240.7	523.4	<0.001 ***	661.1	1077.1	0.540
3	5674.0	557.8	<0.001 ***	6089.3	1713.3	<0.001 ***
4	4444.0	668.1	<0.001 ***	4338.2	1455.5	0.003 **
5	5971.5	7434.0	<0.001 ***	7615.6	1499.9	<0.001 ***
6	5271.5	852.3	<0.001 ***	5412.0	1340.1	<0.001 ***
7	4904.4	1017.8	<0.001 ***	4270.0	1688.5	0.013 *
8	7931.8	1273.7	<0.001 ***	7938.6	2887.5	0.007 **
9	4274.4	1162.5	<0.001 ***	6694.3	1577.1	<0.001 ***
10	5800.8	1330.0	<0.001 ***	5261.5	2161.1	0.016 *
11	2991.8	1599.4	0.061	4146.0	2402.0	0.088
12	5280.6	1618.6	0.001 **	5517.8	3064.2	0.075
13	6325.1	1612.8	<0.001 ***	7702.8	2409.3	0.002 **
14	5332.0	1739.4	0.002 **	5330.3	2470.4	0.034 *
Difference in length of stay						
	Multivariable Linear Regression			Propensity Score Matching		
**Day**	**Estimate**	**SE**	***p*-value**	**Estimate**	**SE**	***p*-value**
1	8.5	1.6	<0.001 ***	5.5	3.2	0.094
2	7.9	1.2	<0.001 ***	5.3	2.7	0.051
3	11.0	1.3	<0.001 ***	13.3	2.8	<0.001 ***
4	8.2	1.5	<0.001 ***	8.9	3.3	0.008 **
5	8.3	1.7	<0.001 ***	11.2	2.2	<0.001 ***
6	10.6	1.9	<0.001 ***	11.8	3.2	<0.001 ***
7	9.4	2.3	<0.001 ***	6.3	4.3	0.147
8	11.5	2.9	<0.001 ***	11.6	4.4	0.010 *
9	13.5	2.6	<0.001 ***	19.8	4.1	<0.001 ***
10	7.6	3.0	0.011 *	7.5	4.1	0.066
11	1.3	3.6	0.726	2.7	3.6	0.464
12	8.6	3.6	0.018 *	10.7	5.2	0.041 *
13	10.9	3.6	0.003 **	13.0	4.7	0.007 **
14	8.7	3.9	0.025	10.9	5.2	0.039 *

* *p* < 0.05; ** *p* < 0.01; *** *p* < 0.001.

**Table 6:** Differences in cost and length of stay for medication events (reference: without event).

Difference in cost						
	Multivariable Linear Regression			Propensity Score Matching		
**Day**	**Estimate**	**SE**	***p*-value**	**Estimate**	**SE**	***p*-value**
1	1429.4	435.2	0.001 **	1570.7	661.7	0.018 *
2	4273.9	473.7	<0.001 ***	2330.0	2834.5	0.412
3	3175.2	651.4	<0.001 ***	3547.6	1402.1	0.012 *
4	878.9	732.3	0.230	1287.4	989.1	0.195
5	5865.1	990.9	<0.001 ***	4504.8	3135.2	0.153
6	7699.2	934.5	<0.001 ***	7086.1	3038.8	0.021 *
7	6762.8	1176.3	<0.001 ***	7387.1	3345.4	0.029
8	3472.6	1240.2	0.005 **	1269.5	2850.6	0.657
9	5358.1	1643.4	0.001 **	7030.1	2676.4	0.011 *
10	2963.4	1522.8	0.052	3854.0	2063.3	0.065
11	5277.3	1581.6	<0.001 ***	−68.5	4087.2	0.987
12	16,128.7	2112.8	<0.001 ***	16,521.5	6651.4	0.016 *
13	6994.6	2096.9	<0.001 ***	8302.9	4628.0	0.078
14	574.9	2029.4	0.777	−1842.1	2317.4	0.430
Difference in length of stay						
	Multivariable Linear Regression			Propensity Score Matching		
**Day**	**Estimate**	**SE**	***p*-value**	**Estimate**	**SE**	***p*-value**
1	3.5	1.0	<0.001 ***	4.4	1.7	0.009 **
2	4.1	1.1	<0.001 ***	4.6	1.8	0.010 *
3	7.4	1.5	<0.001 ***	8.9	3.8	0.020 *
4	1.2	1.7	0.495	0.4	2.9	0.900
5	8.7	2.3	<0.001 ***	6.7	4.3	0.121
6	12.3	2.1	<0.001 ***	11.6	4.1	0.005 **
7	8.9	2.7	<0.001 ***	9.7	4.6	0.038
8	4.3	2.8	0.126	1.5	5.0	0.765
9	8.1	3.7	0.029 *	13.4	4.2	0.002 **
10	0.9	3.4	0.795	4.7	3.4	0.167
11	10.2	3.5	0.004 **	5.0	6.6	0.448
12	12.1	4.7	0.010 *	13.0	8.6	0.135
13	14.9	4.7	0.002 **	21.4	8.3	0.013 *
14	4.6	4.5	0.308	8.7	6.0	0.152

* *p* < 0.05; ** *p* < 0.01; *** *p* < 0.001.

## Supplementary Materials

A total of six raw datasets for analysis in this study are available at Zenodo, accession number: 10.5281/zenodo.3833819. Link: http://doi.org/10.5281/zenodo.3833820.
